# Evaluation of Acute Symptomatic Seizures and Etiological Factors in a Tertiary Care Hospital From a Developing Country

**DOI:** 10.7759/cureus.26294

**Published:** 2022-06-24

**Authors:** Swaapnika Vemulapalli, Anand L Betdur, Ganaraja V Harikrishna, Kavya Mala, Suresha Kodapala

**Affiliations:** 1 Internal Medicine, Vydehi Institute of Medical Sciences and Research Centre, Bangalore, IND; 2 Neurology, Vydehi Institute of Medical Sciences and Research Centre, Bangalore, IND

**Keywords:** epilepsy, status epilepticus, generalized seizures, intracranial tumors, alcohol withdrawal seizures, neuroinfections, cerebrovascular accidents, acute symptomatic seizures

## Abstract

Introduction: The etiologies of acute symptomatic seizures (ASS) differ across the globe. We aimed to evaluate the etiological spectrum of acute seizures and to observe the pattern of seizure types among study participants.

Methodology: We conducted this prospective study from 2016 to 18. We included all patients aged 20 years or older, presenting with ASS. We excluded those with pseudoseizures. We performed appropriate descriptive analyses to describe the demographic details, etiology of ASS, and pattern of ASS.

Results: One hundred and thirty-eight patients were enrolled, constituting about 0.8% of total hospital admissions. The mean age at presentation was 44.33 ± 17.73 years. The most common etiologies for ASS were cerebrovascular accidents (CVA - 32.6%), neuroinfections (26.8%), metabolic derangements (13%), alcohol withdrawal (10.9%), and intracranial tumors (4.3%). 71% of the patients presented with only a single episode of ASS. The predominant type of seizure was generalized tonic-clonic seizures, seen in 70.2% of all patients, followed by focal with the bilateral tonic-clonic type (15.9%) and focal seizures (10.1%). New-onset seizures presenting as status epilepticus were observed in 3.6%.

Discussion: CVA and neuroinfections were the most common causes of ASS in our study, highlighting the need for community awareness of these conditions and the need to seek rapid care. The majority of our patients had only a single episode of seizures, with generalized seizures being the most common type, followed by focal onset seizures.

## Introduction

Acute symptomatic seizures (ASS) are events occurring in close relation to a central nervous system insult. Their causes may be metabolic, toxic, structural, inflammatory, or infectious in origin [1]. The first episode of seizures in an adult is usually a clue to the underlying etiology. Given that it is a medical emergency, determining the etiology is quite important in the management and prevention of seizures. As seizure etiologies are different in developed and developing countries, the common cause of ASS in developing countries is neuroinfections like neurocysticercosis, tuberculosis, and malaria, which are uncommon in developed countries [2]. It also varies with geographic location and is influenced by some endemic diseases which are prevalent in that particular region. There are very few studies from south India regarding the profile of ASS and no recent studies available to understand the profile of ASS in this region. We undertook this study to identify the etiology and pattern of acute seizures in a sample of patients visiting a tertiary care hospital in India, a developing country.

## Materials and methods

This prospective observational study was conducted to evaluate the etiological spectrum of acute seizures and to observe the pattern of seizures among study participants. This study was conducted from 2016 to 2018 at the Vydehi Institute of Medical Sciences and Research Centre, after obtaining approval from the Vydehi Institutional Ethics Committee (reference number PG-EC 2015). All patients aged 20 or more years who presented with ASS were included for analysis. Patients less than 20 years of age, those with pseudoseizures, transient ischemic attacks, eclampsia, and movement disorders like choreoathetosis or tic disorders were excluded. After a detailed history regarding the onset and type of seizure, the number of seizure episodes along with demographic details underlying any medical illness were collected. A complete clinical examination was performed on all patients, and appropriate investigations like CT/MRI brain and electroencephalogram (EEG) were ordered wherever required for confirmation of diagnosis.

All analyses were carried out using SPSS 20.0 (IBM Corp, Armonk, NY). A mean with standard deviation (SD), and range were used to express continuous variables, while percentages were used for categorical variables.

## Results

One hundred and thirty-eight patients (92 males and 46 females) were included, representing about 0.8% of the total hospital admissions during the study period. The mean (SD) age at presentation was 44.33 ± 17.73 years. Most of the patients were aged between 21 and 30 years, constituting 32.6% of the study population. Common causes of seizures were CVA, seen in 32.6% of our sample, followed by neuroinfections (26.8%), metabolic derangements (13%), and alcohol withdrawal seizures (10.9%). In about 10.1% of patients, the cause of seizures was classified as idiopathic, as it could not be identified even after extensive investigations (Table 1). None of these patients were on seizure medication prior.

**Table 1:** Etiological profile of acute symptomatic seizures observed in study population.

Etiology		Number	Percentage (%)
Cerebrovascular accident		45	32.6
	Ischemia	22	15.9
	Hemorrhage	16	11.6
	Scar epilepsy (old cerebrovascular accident)	5	3.6
	Cortical venous thrombosis	2	1.4
Neuroinfections		37	26.8
	Neurocysticercosis	20	14.5
	Neurotuberculosis	12	8.7
	Meningoencephalitis	4	2.9
	Encephalitis	1	0.7
Metabolic derangements		18	13
	Uremia	5	3.6
	Hypoglycemia	5	3.6
	Hyperglycemia	4	2.9
	Hypocalcemia	2	1.5
	Hyponatremia	2	1.5
Alcohol withdrawal seizures		15	10.9
Intracranial tumors		6	4.3
	Glioma	3	2.2
	Meningioma	1	0.7
	Secondaries	2	1.4
Idiopathic		14	10.1
Miscellaneous		3	2.1
Total		138	100

Among males, the most common cause of ASS was CVA, seen in 34.7%, followed by neuroinfections, seen in 23.9%. Among females, common etiologies were neuroinfections, seen in 32.6%, followed by CVA, seen in 28.2%.

**Figure 1:**
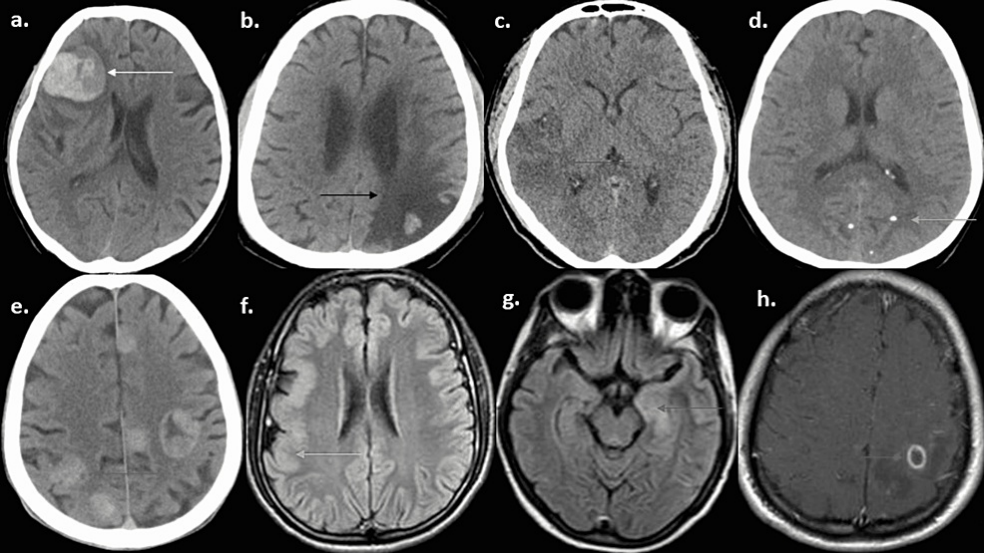
Computed tomography brain axial sequences showing (a) intracranial hemorrhage (white arrow), (b) chronic infarct in left parietal-occipital region (black arrow), (c) acute infarct in right parietal region (red arrow), (d) calcified stage of neurocysticercosis (orange arrow), (e) multiple brain metastases (green arrow). MRI T2 FLAIR axial sequences showing (f) focal cortical dysplasia in right hemisphere (yellow arrow), (g) bilateral mesial temporal hyperintensities in herpes encephalitis (purple arrow). MRI T1 post-contrast sequence showing (h) ring-enhancing lesion of tuberculoma (blue arrow). MRI: magnetic resonance imaging, FLAIR: fluid-attenuated inversion recovery.

In patients with CVA, ischemic strokes were the most common, seen in 15.9%. Among those with neuroinfections, 14.5% of patients had neurocysticercosis and 8.7% of patients had neurotuberculosis - either tuberculoma (7.2%) or tuberculous meningoencephalitis (1.5%). Metabolic derangement was the cause of ASS in 13% of patients, with uremia and hypoglycemia being the most common (3.6% each). Other causes included intracranial tumors (4.3%), acute kidney injury (0.7%), focal cortical dysplasia (0.7%), and progressive multifocal leukoencephalopathy (0.7%). Representative images of various etiologies are given in Figure 1.

In our study, 71% of patients presented with only one episode of ASS, with the rest having more, particularly those with neurocysticercosis and neurotuberculosis. The predominant type of ASS was generalized onset seizure (70.2%) of patients, followed by focal with the bilateral tonic-clonic type (15.9%) and focal seizures (10.1%) based on the 2017 International League Against Epilepsy (ILAE) classification of epilepsies. The most common cause of generalized seizures was CVA (26.8%), followed by metabolic derangement (12.3%). Among patients with focal seizures, the most common cause was neuroinfections (7.2%). Neuroinfections (10.8%) were the most common cause of ASS in those with focal seizures with generalization (Table 2).

**Table 2:** Types of seizures in the study population.

Types of seizure		No. of patients (n=138)	Percentage (%)
Generalized tonic-clonic		97	70.2
	Cerebrovascular accidents	37	26.8
	Neuroinfections	09	6.5
	Alcohol withdrawal	15	10.8
	Metabolic	17	12.3
	Tumor	06	4.3
	Idiopathic	12	8.6
	Miscellaneous	01	0.72
Focal		14	10.1
	Cerebrovascular accidents	03	2.1
	Neuroinfections	10	7.2
	Miscellaneous	01	0.72
Focal with bilateral tonic clonic		22	15.9
	Cerebrovascular accidents	03	2.1
	Neuroinfections	15	10.8
	Metabolic	01	0.72
	Idiopathic	02	1.5
	Miscellaneous	01	0.72
Status epilepticus		05	3.6
Total		138	100

Among various causes of ASS, CVA was more common in the elderly population with a mean age at presentation of 58.32 ± 23.18 years (range: 35-81 years), while neuroinfections were common in the younger population at 29.65 ± 19.25 years (range: 19-49 years). A total of 3.6% of patients presented with status epilepticus, secondary to CVA (1.5%) and neuroinfections (2.1%).

## Discussion

We describe our experience with ASS in India, a developing country. Overall, we found CVA to be the most common cause, followed by neuroinfections. However, neuroinfections were more commonly observed in the younger population than CVA which was common in the older population. Identification of the cause of seizures is of prime importance, as it has a bearing on the nature of the treatment, follow-up, and outcome of each case.

We had 138 cases during the study period, which constituted 0.8% of the total admissions. An international study done in Italy showed that the incidence of acute symptomatic seizures is 6.3%, which is higher compared to our study [3]. Bleck et al. noted that 3.5% of patients with critical medical illness had new-onset seizures [4]. ASS is more commonly seen at the extremes of age, probably due to the increased incidence of metabolic, infectious, and encephalopathic disorders in infants and an increased incidence of tumors and cerebrovascular disease in the elderly [5]. Unlike other studies, we did not include pediatric patients, which could have contributed to the relatively lower incidence of ASS.

We found that ASS was more common among males, with about two-thirds of our sample being males. This male preponderance could be due to the greater number of alcohol withdrawal seizures, which we observed exclusively among males in our sample. In India, alcohol consumption is largely restricted to males, particularly among the lower socioeconomic strata. Similar sex ratios have been reported in previous studies [6-8].

In our study, CVA was the most common cause of ASS, followed by neuroinfections. Another study from Egypt also found that CVA was the most common identifiable cause of seizures, followed by idiopathic epilepsy and metabolic causes [9]. However, in comparison to developed countries, we found a higher incidence of ASS due to CVA [3]. In Italy, researchers observed hemorrhagic stroke and cortical lesions were independent predictors of ASS [3]. This lower incidence probably could be due to greater and rapid access to necessary interventions, compared to developing countries like ours - in CVA, particularly in hemorrhagic strokes, the risk of ASS increases with time [10].

In one study from south India, neuroinfection was the leading cause of ASS, seen in 36%, with the majority of neuroinfection being neurocysticercosis [11]. This difference with ours is likely attributable to the endemicity of different infections in different parts of India. In a study from western India, which included patients older than 40 years, the most common type of ASS was generalized tonic-clonic seizures, with the most common cause being CVA, as seen in our study [12]. Similarly, in east India, CVA was again the most common etiology, and generalized tonic-clonic seizures were the most common type [13]. However, in both these studies, the vast majority of patients were older than 60 years, unlike ours [12,13]. In central India, the authors found neuroinfections (31%) to be the leading cause of new-onset ASS, which mainly presented as a focal seizure. CVA (26%) was the second most common cause, mainly presenting as generalized seizures [14]. Finally, in a large study from south India, the authors found that 2.4% of their hospital admissions were related to new-onset ASS, with the most common etiologies being neuroinfections (32%), metabolic disorders (32%), and CVA (21%) [15]. Thus, from India at least, neuroinfections, metabolic disorders, and CVA appear to be the most common causes of ASS, with variations probably resulting due to endemicity and other local factors. In Nigeria too, neuroinfections and CVA were the prominent causes of ASS, with infective causes more common among those younger than 50 years old and CVA more common in those older than 50 years [16]. In Singapore, a developed country, the most common risk factor for ASS, especially in the elderly, was CVA (30-70%), with tumors relatively more frequent (10-15%) [17]. Thus, it is fair to say that CVA remains the most common etiologic factor for ASS across the globe, with other etiologies varying between developed and developing countries.

Overall, although there is significant heterogeneity in the etiology and age at presentation, generalized seizures were the most common form of ASS across studies, as seen in our study as well [18]. This, in our clinical experience, could be due to caretakers' perceiving these seizures as more severe, thus prompting rapid travel to a healthcare facility. Among those with status epilepticus, the predominant cause in our sample was neuroinfections, also similar to previous studies [16,18].

Ours is one of the largest studies on ASS from the region and provides valuable information for internists, neurologists, and epileptologists on the pattern of ASS in a developing country. However, our study has limitations. We did not follow up on the cases to study their outcomes, and we excluded pediatric cases. This could have provided a more comprehensive overview of the pattern of ASS.

## Conclusions

The vast majority of ASS in our sample occurred in relatively young patients and as a single episode. The most common causes were CVA and neuroinfections. Our findings highlight the need for increased community health awareness and preventative education, as well as the need for a greater degree of suspicion of these conditions among clinicians treating patients presenting with ASS.
